# Identification of Rare Genetic Variants in Familial Spontaneous Coronary Artery Dissection and Evidence for Shared Biological Pathways

**DOI:** 10.3390/jcdd10090393

**Published:** 2023-09-12

**Authors:** Tamiel N. Turley, Jeanne L. Theis, Jared M. Evans, Zachary C. Fogarty, Rajiv Gulati, Sharonne N. Hayes, Marysia S. Tweet, Timothy M. Olson

**Affiliations:** 1 Molecular Pharmacology and Experimental Therapeutics Track, Mayo Clinic Graduate School of Biomedical Sciences, Mayo Clinic, Rochester, MN 55905, USA; turley.tamiel@mayo.edu; 2 Cardiovascular Genetics Research Laboratory, Mayo Clinic, Rochester, MN 55905, USA; theis.jeanne@mayo.edu; 3 Department of Quantitative Health Sciences, Division of Computational Biology, Mayo Clinic, Rochester, MN 55905, USA; jaredmevans@outlook.com (J.M.E.); fogarty.zachary@mayo.edu (Z.C.F.); 4 Department of Cardiovascular Medicine, Mayo Clinic, Rochester, MN 55905, USA; gulati.rajiv@mayo.edu (R.G.); hayes.sharonne@mayo.edu (S.N.H.); tweet.marysia@mayo.edu (M.S.T.); 5 Department of Pediatric and Adolescent Medicine, Division of Pediatric Cardiology, Mayo Clinic, Rochester, MN 55905, USA

**Keywords:** acute coronary syndrome, collagen genes, co-segregation analysis, spontaneous coronary artery dissection, myocardial infarction, whole-genome sequencing

## Abstract

Rare familial spontaneous coronary artery dissection (SCAD) kindreds implicate genetic disease predisposition and provide a unique opportunity for candidate gene discovery. Whole-genome sequencing was performed in fifteen probands with non-syndromic SCAD who had a relative with SCAD, eight of whom had a second relative with extra-coronary arteriopathy. Co-segregating variants and associated genes were prioritized by quantitative variant, gene, and disease-level metrics. Curated public databases were queried for functional relationships among encoded proteins. Fifty-four heterozygous coding variants in thirteen families co-segregated with disease and fulfilled primary filters of rarity, gene variation constraint, and predicted-deleterious protein effect. Secondary filters yielded 11 prioritized candidate genes in 12 families, with high arterial tissue expression (*n* = 7), high-confidence protein-level interactions with genes associated with SCAD previously (*n* = 10), and/or previous associations with connective tissue disorders and aortopathies (*n* = 3) or other vascular phenotypes in mice or humans (*n* = 11). High-confidence associations were identified among 10 familial SCAD candidate-gene-encoded proteins. A collagen-encoding gene was identified in five families, two with distinct variants in COL4A2. Familial SCAD is genetically heterogeneous, yet perturbations of extracellular matrix, cytoskeletal, and cell–cell adhesion proteins implicate common disease-susceptibility pathways. Incomplete penetrance and variable expression suggest genetic or environmental modifiers.

## 1. Introduction

Spontaneous coronary artery dissection (SCAD) has gained recognition over the past decade as an important cause of acute coronary syndrome, myocardial infarction, and sudden death in predominantly young to middle-aged women with few atherosclerotic risk factors [1]. Complete or partial occlusion of the coronary vessel lumen by an expanding medial hematoma and/or an intimal tear are the diagnostic features of SCAD on coronary angiography. Coexistence of fibromuscular dysplasia (FMD) in >50% of individuals with SCAD [1], identification of a shared genetic risk allele for FMD and SCAD [2], and association with non-FMD extra-coronary aneurysms or dissections suggest that SCAD is a clinical manifestation of a systemic arteriopathy. Although a majority of cases are sporadic, the lack of clinical risk factors for vascular disease together with reports of rare familial associations implicate a genetic predisposition to SCAD [3]. In a Mayo Clinic cohort, whole-genome sequencing (WGS) and genome-wide associations studies (GWAS) previously revealed a rare co-segregating variant in TLN1 with major effect in a familial case, and common risk-conferring variants in sporadic cases, respectively [4,5,6]. In a large cohort from the United Kingdom, WGS and adjudication of rare variants by American College of Medical Genetics (ACMG) criteria revealed seven genes that harbored pathogenic or likely-pathogenic variants, yet they represented only 3.6% of cases [7]. Indeed, SCAD is becoming increasing recognized as a genetically heterogeneous and complex disorder, necessitating comprehensive and multidisciplinary approaches for discovery and validation of susceptibility genes [8]. In the current study, WGS in 15 families provided a unique opportunity to identify rare co-segregating variants and candidate genes to advance understanding of SCAD pathogenesis.

## 2. Materials and Methods

### 2.1. Study Subjects

Probands from each family were consecutively enrolled in the Mayo Clinic SCAD registry after diagnostic confirmation of SCAD by review of coronary angiograms and abstraction of detailed demographic and clinical data from questionnaires and medical records from 30 August 2011 to 30 June 2020 [9]. The diagnostic criteria for SCAD were as previously described [5,10]. First-degree and more distant relatives with a clinical diagnosis of SCAD or other arteriopathy, which included known FMD, arterial aneurysm or dissection, and cerebral infarctions, were invited to participate in the study.

### 2.2. Next-Generation Sequencing and Bioinformatics Analysis

WGS and whole-exome sequencing (WES) were performed by the Mayo Clinic Medical Genome Facility, and variant call format (VCF) files were created by the Bioinformatics Core. For WGS of 15 probands and 22 family members, 150 base pair (bp) paired-end sequencing was performed on either the Illumina HiSeq4000 or NovaSeq 6000 (Illumina, San Diego, CA, USA). For 10 family members, whole-exome 100 bp or 150 bp paired-end sequencing was performed on either the Illumina HiSeq2000 or 4000 platforms, respectively, using the Agilent SureSelect Human All Exon capture kits (V4 + UTR or V5 + UTR; Agilent technologies, Santa Clara, CA, USA).

WGS and WES reads were aligned to the hg38 or hg19 reference genome with either BWA-MEM [11] or Novoalign [12], respectively. Alignments were followed by the sorting and marking of duplicate reads using Picard [13]. Local realignment of insertions/deletions (INDELs) and base quality score recalibration were performed using the Genome Analysis Toolkit version 3.4 [14]. Single-nucleotide variants (SNVs) and INDELs were called across all samples simultaneously using the Genome Analysis Toolkit’s UnifiedGenotyper [15] (WES reads) or GATK HaplotypeCaller [16] and GenotypeGVCFs [14] (WGS reads), followed by variant quality score recalibration. WGS yielded on average 1.1 billion 150 base paired-end reads, and WES yielded 101 million 100- and 150-base paired-end reads.

### 2.3. Genetic Variant Filtering and Prioritization

VCF files with SNV and INDEL calls from WGS and WES were uploaded into Ingenuity^®^ Variant Analysis™ (IVA; QIAGEN, Redwood City, CA, USA). To retain only high-confidence variants, a quality control filter was applied that required a base call quality of at least 20, genotype quality of ≥30, and a read depth of ≥10. Variants within a simple repeat region, defined as 1–50 bp in unit length with ≥5 repetitions, were excluded using the Tandem Repeats Finder [17] algorithm.

Annotated variants that passed quality control were prioritized by filtering for rarity, evidence of co-segregation with disease, impact on protein, tolerance of the corresponding gene to variation, mRNA expression pattern in arterial tissues, and gene–disease association. Variants with a minor allele frequency (MAF) of <0.1% from the Genome Aggregation Database [18] (gnomAD) v2.1 and v3 across all races were included. Variants with an MAF of <1% were used for recessive inheritance modeling. Co-segregation with disease required that a variant was present in all affected family members and obligate carriers. Except for parents of SCAD probands, unaffected family members were not included in the study. Co-segregation modeling was based on autosomal dominant transmission of a heterozygous variant with incomplete penetrance and variable expression and/or inheritance of homozygous or compound heterozygous recessive alleles for the affected sibling cases. 

To predict the consequence of SNVs and INDELs, coding variants were annotated utilizing the Ensembl Variant Effect Predictor [19] tool. Variants classified as missense, frameshift, start-loss, stop-gain, stop-loss, and canonical splice site loss variants were included. The predicted deleterious effect of missense and canonical splice site variants was evaluated using a Combined Annotation Dependent Depletion [20] (CADD) score, which was then indexed as a percentile of genome-wide variants according to variant type. CADD percentiles were computed using a custom script that generated a histogram of CADD PHRED scores for all variants with the same variant consequence. Missense and canonical splice site variants each with a CADD score of ≤24.5, corresponding to the lower quartile, were excluded. Conservation of substituted amino acids was determined from the NCBI HomoloGene database and multialigned with MUSCLE version 3.6 [21]. The IVA software platform was used to identify and prioritize variants that were classified as deleterious by the Human Gene Mutation Database (HGMD) [22].

Non-coding variants were predicted to impact gene regulation based on location within a microRNA (miRNA) binding/coding site or a transcription factor binding site (TFBS) with a Position Weight Matrix score >0.75 from the Factorbook database [23]. Variants were prioritized by filtering for CADD score ≥7 corresponding to the upper quartile specific to regulatory variants, and rank score of ≤3 in the RegulomeDB V2.0 database [24]. To eliminate false positives, in silico visual assessment of all variants was performed using the Integrative Genomics Viewer [25].

### 2.4. Candidate Gene Prioritization

Publicly available databases and in silico tools were used to predict the biological impact of each identified variant. For a coding variant, the gene’s intolerance to variation was determined by metrics in gnomAD [18]. A probability of loss-of-function (pLI) and/or an observed over expected (o/e) score applied to stop gain/loss and frameshift variants, and a Z score applied to missense and canonical splice site variants. All candidate genes were required to have a pLI score ≥0.9 (range 0.0–1.0) and/or an o/e of <0.35, or a Z score ≥ 2. Regulatory variants were required to be predicted as pathogenic with a score ≥0.5 according to the Functional Analysis through Hidden Markov Models with eXtended Features (FATHMM-XF) V2.3 database [26].

Secondary filters to prioritize genes that harbored rare, predicted-deleterious, co-segregating variants were based on high arterial tissue expression ranking, a candidate gene shared by two or more familial SCAD (F-SCAD) cases, known or predicted functional associations with SCAD candidate genes previously identified in our cohort (TLN1, EDN1, PHACTR1, ADAMTSL4, C1orf54, ECM1, FBN1, LINC00310, LRP1, MRPS21, AFAP1) [2,4,5,6], an established gene for monogenic connective tissue disorders or aortopathies, or a gene associated with vascular disease risk. High arterial tissue messenger ribonucleic acid (mRNA) expression was defined by 1–5 ranking for one or more arterial tissues (aorta, coronary, or tibial), among 54 distinct tissue types in the Genotype-Tissue Expression (GTEx) database [27]. Protein–protein interaction networks were generated with the STRING v11 database [28] and visualized with Cytoscape v3.8.2 software [29]. The commercially available Heritable Disorders of Connective Tissue Panel (57 genes (2019); GeneDx, Gaithersburg, MD, USA) and Aortopathy Comprehensive Panel (27 genes (2019); Invitae, San Francisco, CA, USA) were used to identify known disease genes. A link to a vascular disease phenotype was identified based upon (1) a genetic mouse model reported in Mouse Genome Informatics (MGI) [30], (2) an established genetic link to vascular disease in humans reported in HGMD [22], or (3) a vascular disease-associated trait annotated in the GWAS Catalog [31]. The secondary filtering process was non-iterative, and top candidate gene(s) selection for each family was based on fulfilling the highest number of secondary filtering parameters.

### 2.5. In Silico Analysis of Shared Biological Networks and Processes

A network propagation analysis was performed on 11 prioritized candidate genes using STRING [28] v11 with a high-confidence threshold of 0.7 and visualized with Cytoscape [29] v3.8.2 to identify shared predicted and known functional associations among F-SCAD candidate genes. The network was built by inputting the previously reported F-SCAD gene TLN1 [4], known TLN1-interacting genes (18 alpha-integrins, 9 beta-integrins, and 7 actin isoforms), and the prioritized candidate genes. Interactions included both direct (physical) and indirect (functional) associations with high-confidence thresholds of >0.7. Enriched functional pathways and gene ontologies were investigated using biological molecular databases that included the Kyto Encyclopedia of Genes and Genomes [32] (KEGG), Reactome [33], National Center for Biotechnology Information BioSystems [34], and GeneALaCart meta-analysis tool from GeneCards [35].

## 3. Results

### 3.1. Clinical Characteristics

**Figure 1:**
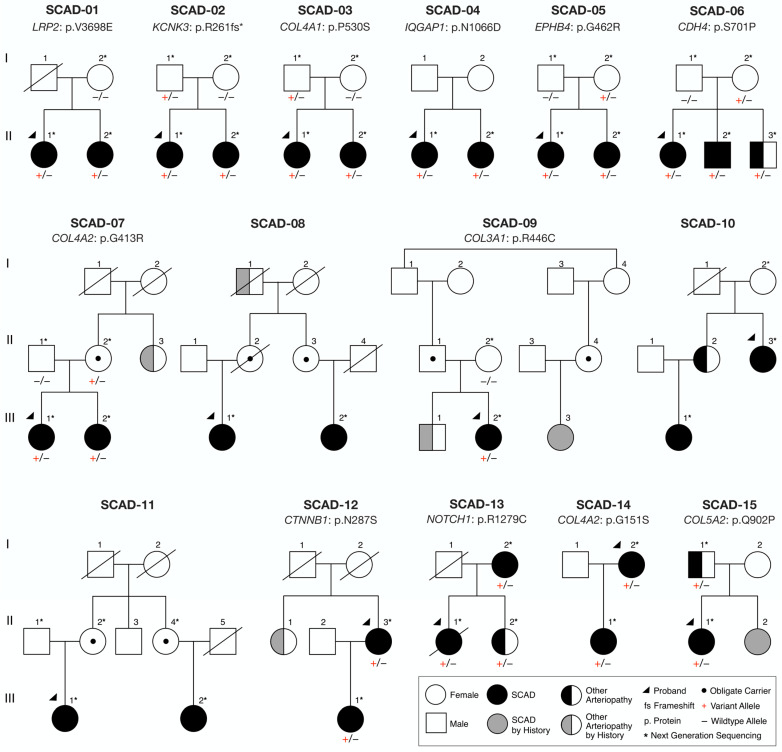
Familial SCAD cohort and prioritized candidate genes. Candidate genes: *CDH4*, cadherin 4; *COL3A1*, collagen type III alpha I chain; *COL4A1*, collagen type IV alpha I chain; *COL4A2*, collagen type IV alpha 2 chain; *COL5A2*, collagen type V alpha 2 chain; *CTNNB1*, catenin beta 1; *EPHB4*, ephrin type-B receptor 4; *IQGAP1*, IQ-motif-containing GTPase-activating protein 1; *KCNK3*, potassium channel subfamily K member 3; *LRP2*, LDL-receptor-related protein 2; *NOTCH1*, neurogenic locus notch homolog protein 1.

Clinical data and DNA samples were obtained from fifteen probands with SCAD who had a relative with SCAD, eight of whom had a second relative with extra-coronary arteriopathy (Figure 1, Table 1). Families were comprised of two affected mother–daughter pairs (SCAD-12, 14), two affected sister pair–parent trios (SCAD-13, 15), one affected sibling trio (SCAD-06), six affected sister pairs (SCAD-01, 02, 03, 04, 05, 07), one affected sibling pair–daughter trio (SCAD-10), and three families with distantly-related affected relatives (SCAD-08, 09, 11).

Among the 15 familial SCAD (F-SCAD) kindreds, SCAD was confirmed by review of angiograms or medical records (*n* = 28) or reported by history (*n* = 2). Probands and relatives with SCAD had a mean age at first event of 43.4 ± 7.2 years (range 32 to 59 years), with the left anterior descending coronary artery branch most affected. Associated phenotypes included FMD of the renal, iliac, splenic, or carotid arteries in twelve individuals who were screened; coronary artery tortuosity in five; peripartum status in three; and migraine headaches in five. Probands in three families had recurrent SCAD in a location separate from the initial event, occurring 2–11 years after the previous dissection. Recent episodes of emotional stress were reported in seven individuals and physical exertion in three individuals as potential triggers for SCAD. Similar to findings in previous studies of SCAD, typical risk factors for atherosclerotic coronary artery disease were less frequent in F-SCAD. Only five individuals had hypertension, nine had hyperlipidemia, eight had active or prior tobacco use, and one had diabetes mellitus. Overall, demographic and clinical characteristics of our F-SCAD cases were indistinguishable from our previously reported SCAD cohort (484 individuals) [5], with the exception of the proportion with migraine headaches (36% vs 15%; *p* = 0.019) and hypertension (32% vs 15%; *p* = 0.038). A majority of patients with SCAD or another arteriopathy were women (34 of 38; 89%), all of white European descent, with a mean age at diagnosis of 44.9 ± 10.1 years (range 25 to 71 years). The phenotypes of relatives with other arteriopathies (*n* = 8) included abdominal, iliac, and/or cerebral aneurysms; carotid or superior mesenteric artery dissections; coronary tortuosity; and stroke.

### 3.2. Whole-Genome Sequencing Reveals Disease-Associated Candidate Genes in Familial SCAD

WGS was performed on genomic DNA samples from affected individuals (*n* = 31) in each of the 15 families (Figure 1). Unaffected parents underwent WGS or WES (*n* = 16) to determine transmission of identified candidate variants. All genome and exome sequencing passed standard quality control metrics for coverage. Variant call format files were uploaded and analyzed for each individual with Ingenuity Variant Analysis software.

**Figure 2:**
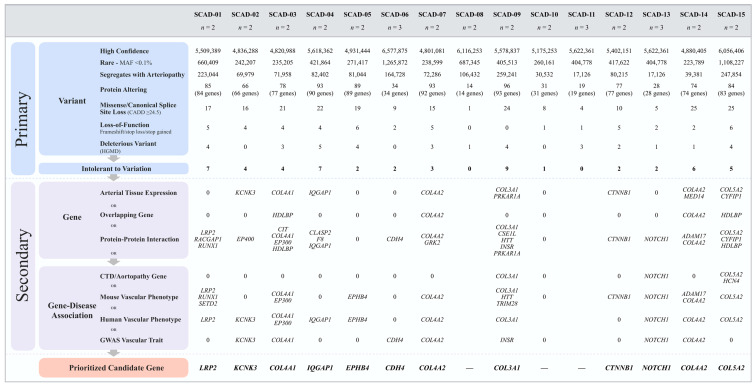
Genetic variant filtering and gene prioritization workflow identified top candidate genes for familial SCAD. A variant filtering scheme was applied to all single-nucleotide variants and insertions/deletions, identifying 11 genes in 12 families. Top candidate gene(s) selection for each family was based on fulfilling the highest number of secondary filtering parameters. Abbreviations: CADD, Combined Annotation Dependent Depletion; CTD, connective tissue disorder; GWAS, genome-wide association study; HGMD, Human Gene Mutation Database; MAF, minor allele frequency; pLI, probability of loss-of-function; SCAD, spontaneous coronary artery dissection.

**Figure 3:**
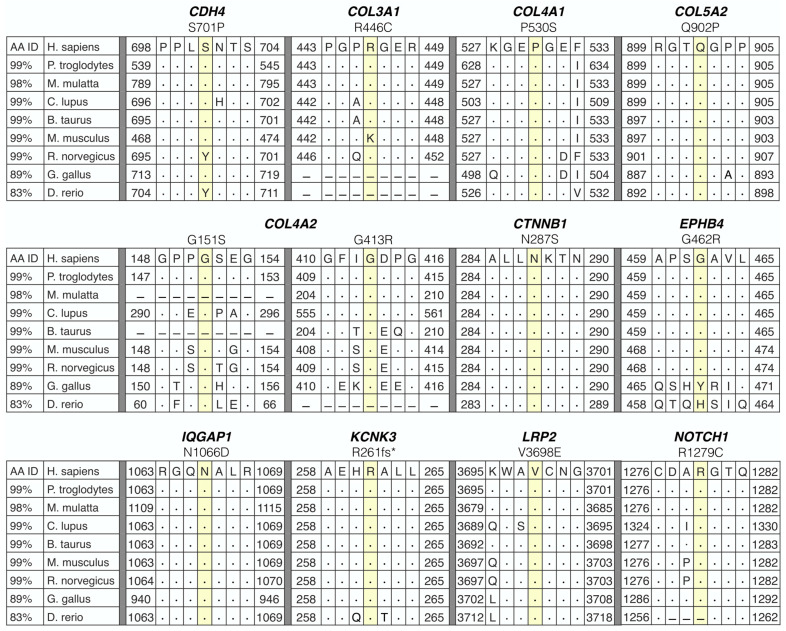
Predicted-damaging variants in familial SCAD altered highly conserved amino acids. Alignment of variants and surrounding residues demonstrated conservation across species. Identical residues are indicated by a dot, and a dash indicates a gap.

For each of the 15 families, over six million variants in 55,000 genes were identified. A systematic filtering approach based on quantitative metrics was applied to identify the most plausible candidate gene(s) for F-SCAD in 15 kindreds (Figure 1 and Figure 2). The primary variant-level filter yielded 54 candidate variants among families, which were further scrutinized by a secondary gene and disease-level filter. The 12 identified variants within each of the 11 prioritized candidate genes were heterozygous nucleotide substitutions or deletions that resulted in amino acid substitutions or frameshifts, all within highly conserved regions (Figure 3). All variants fulfilled quality control metrics, were rare in the general population with a minor allele frequency of <0.1% (gnomAD), co-segregated with disease; and were predicted deleterious based on CADD score, loss of function variant, and/or HGMD link (Table 2). 

**Figure 4:**
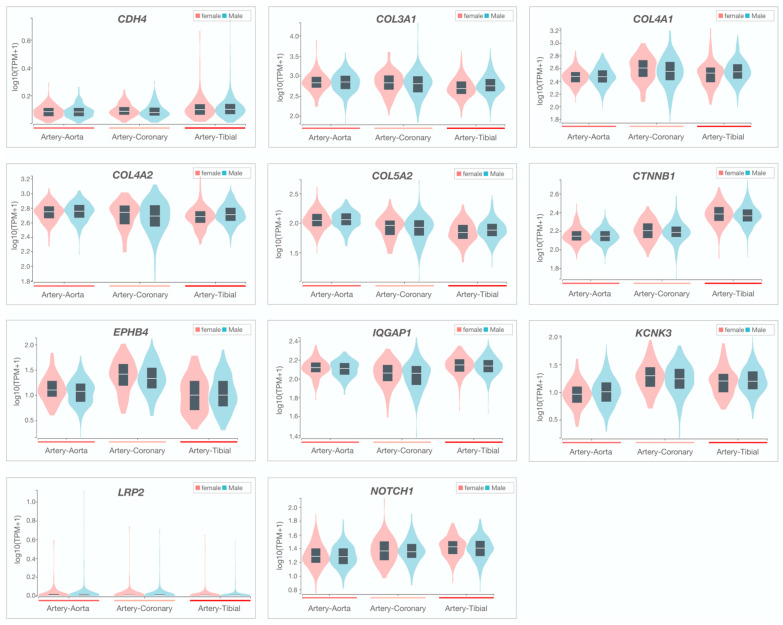
Sex-specific arterial tissue expression of prioritized candidate genes. Violin plots were generated by GTEx, demonstrating no differences in sex-specific candidate gene expression in coronary, aorta, and tibial arteries.

At the gene level, candidates were required to be intolerant to variation with a probability of loss-of-function score >0.9 and/or an observed over expected score of >0.35 for truncating variants, or a Z-score of >2 for missense variants. For each family, 0–9 candidate genes fulfilled all primary variant and gene-constraint metrics. Among the six families with two or more affected siblings, no homozygous or compound heterozygous variants were identified that passed the primary filters, excluding autosomal recessive inheritance. All families were thus considered to have autosomal dominant inheritance of a heterozygous variant with incomplete penetrance and, in families with extra-coronary arteriopathy, variable expression. A secondary filter of seven additional gene- and disease-level metrics was applied to identify the top candidate gene(s) in each family. Seven genes ranked highly in arterial tissue expression (rank 1–5) among 54 distinct tissues, none of which showed differential expression in females (Table 2, Figure 4).

**Figure 5:**
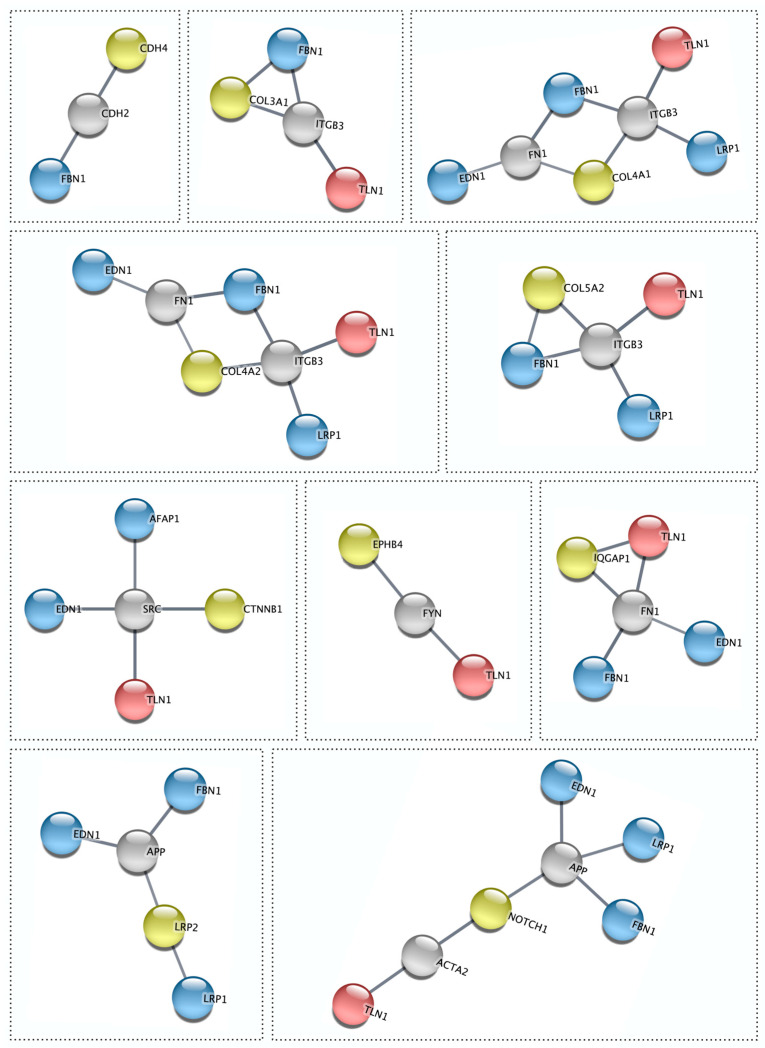
Functional associations between individual F-SCAD candidates and previously reported SCAD genes. Talin 1 (red) and/or GWAS-derived candidate gene proteins EDN1, LRP1, FBN1, and AFAP1 (blue) interacted with 10 of the prioritized candidates for familial SCAD (yellow) directly or through a STRING second shell interactor protein (grey). STRING analysis was set at a high confidence score (0.7).

Our previous genomic studies of familial and sporadic SCAD revealed TLN1, FBN1, LRP1, PHACTR1, ECM1, ADAMTSL4, LINC00310, C1orf54, MRPS21, EDN1, and AFAP1 as candidate genes for disease susceptibility [4,5,6]. Accordingly, potential protein–protein interactions of these 11 genes with the 11 F-SCAD candidate genes identified in the current study were investigated by STRING, revealing 10 high-confidence first- or second-degree functional associations. Four high-confidence first-degree interactions were identified: IQGAP1–TLN1, COL3A1–FBN1, COL5A2–FBN1, and LRP1–LRP2. Two of the previously identified SCAD candidate genes—TLN1 and FBN1—each had eight high-confidence first- and/or second-degree interactions (Figure 5, Table 2). 

To further determine a gene’s candidacy, a biological context filter was applied to identify genes that were previously associated with aortopathies and connective tissue disorders (CTD). This comprehensive analysis revealed three genes that overlapped both CTD and aortopathy gene panels (COL3A1, COL5A2, NOTCH1; Table 2). Candidate genes were scrutinized for associations with reported murine or human vascular abnormalities in the Mouse Genome Informatics and HGMD databases or a GWAS vascular trait in the GWAS Catalog. From this analysis, 11 genes with known vascular phenotype-gene associations were identified. Overall, 12 heterozygous nucleotide substitutions or deletions that resulted in amino acid substitutions or frameshifts within highly conserved regions of 11 genes passed the systematic filtering metrics.

### 3.3. Potential Associations among Familial SCAD Candidate Genes

**Figure 6:**
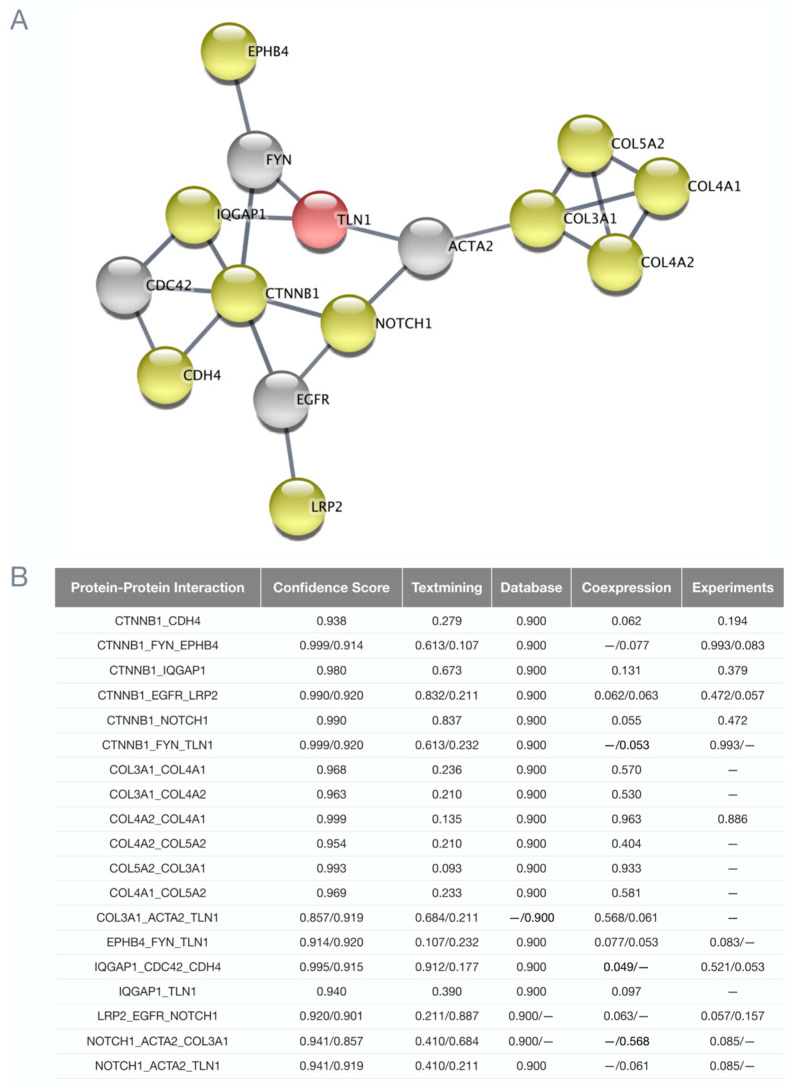
Network interactome of functional associations among F-SCAD candidates. (**A**) Protein–protein interactions in 10 of the familial prioritized candidates (yellow) and talin 1 (red) directly or a STRING 2nd shell interactor (grey). (**B**) Shared protein–protein interactions had a high confidence score of <0.9.

For five of the twelve families, the top candidate was a collagen-encoding gene, each of which fulfilled five or more secondary filtering parameters. Moreover, two families had distinct heterozygous missense variants in COL4A2, which encodes the collagen type IV alpha 2 chain (SCAD-07 and SCAD-14; Figure 1, Table 2). To identify additional associations among F-SCAD candidate genes, interactions at the protein level were investigated by creating a protein–protein network of TLN1 and the 11 other candidate gene proteins in STRING. Among the 11 top-ranked F-SCAD candidates, 10 genes had high-confidence, first- or second-degree associations with another F-SCAD-candidate-gene-encoded protein (Figure 6).

**Figure 7:**
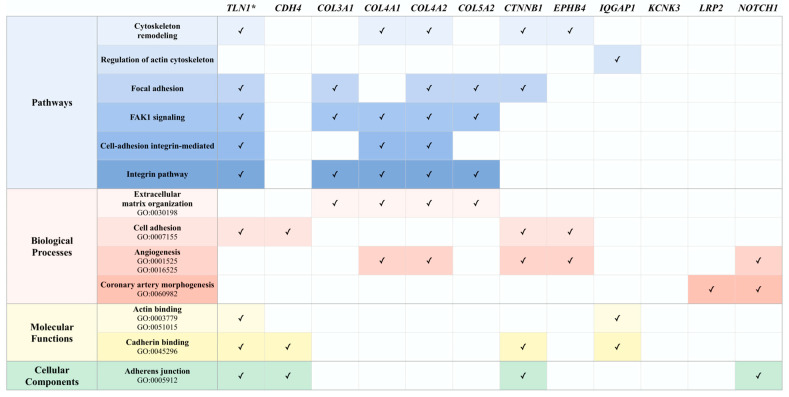
Familial candidate genes share pathways and biological processes involved in the cytoskeleton and angiogenesis. Abbreviations: FAK1, focal adhesion kinase 1; GO, Gene Ontology. Asterisks denotes the previously identified *TLN1*.

Genetic perturbation of the actin cytoskeleton has been proposed as one pathway in SCAD pathogenesis [4,5,6]. Accordingly, shared pathways and biological processes of the actin cytoskeleton and blood vessel formation were investigated using curated pathway and Gene Ontology databases. Among the 11 candidate genes, 10 genes shared pathways and processes involved in the cytoskeleton structure, remodeling and signaling, and/or angiogenic processes (Figure 7).

### 3.4. Ancillary Investigation of Non-Coding Variants

Our comprehensive and stringent variant filtering and gene prioritization scheme identified a top-ranked candidate gene in 12 of the 15 families in analyses confined to variants in coding regions that altered protein structure. To explore the possibility that rare non-coding variants predicted to alter gene expression could co-segregate with SCAD, an ancillary investigation of defined regulatory domains was conducted in the three families in which a candidate gene was not identified. Following the same primary analysis used for coding variants, implementation of secondary filters identified rare heterozygous variants within a microRNA binding site in PDGFRA (SCAD-08) and transcription factor binding site in ATF4 (SCAD-10) (Figures S1 and S2, Table S1). Both candidates demonstrated second-degree protein interactions with both previously identified SCAD-associated genes and other F-SCAD candidate genes with coding variants identified in the current study (Figures S3 and S4). 

## 4. Discussion

SCAD is becoming increasingly recognized as a complex, genetically heterogeneous disorder [8]. To discover susceptibility genes for SCAD and potential shared disease pathways, we performed WGS in 15 rare F-SCAD cases and applied a systematic filtering approach that utilized quantitative metrics at variant, gene, and disease levels. Consistent with the low yield of pathogenic variants identified in a large sporadic SCAD cohort [7], only two top-ranked variants in NOTCH1 and KCNK3 in the current study were classified as pathogenic by ACMG criteria. However, co-segregation with SCAD and extra-coronary arteriopathy proved to be a powerful prioritization filter, reducing the number of rare, predicted-deleterious variants by 36–96% among families. Stringent gene constraint thresholds were used to identify variants with the highest likelihood of perturbing protein function, rendering short lists of 0–9 candidate genes in each family. Further prioritization of these genes was based on arterial tissue expression, interaction with known SCAD-associated genes, and genetic association with murine and human vascular disorders. The 12 identified heterozygous nucleotide substitutions and deletions exhibited autosomal dominant inheritance with incomplete penetrance and variable expression, including in the six families with 2–3 affected siblings for which no variants fitting an autosomal recessive mode of inheritance were identified. Collagen-encoding genes were the most common class among the 11 top-ranked candidate genes in our F-SCAD cohort, a finding consistent with an emerging body of evidence from WES and WGS in sporadic SCAD cohorts [36,37,38].

Three of the top-ranked F-SCAD candidate genes (COL3A1, COL5A2, and NOTCH1) are disease genes for inherited aortopathy and CTDs. While FMD is the most common trait associated with SCAD, 5.1–8.2% of sporadic SCAD cases have been found to carry deleterious variants within genes associated with heritable CTDs and aortopathy, including vascular Ehlers–Danlos syndrome (COL3A1), Marfan syndrome (FBN1), and Loeys–Dietz syndrome (SMAD3) [7,39,40]. Accordingly, familial variants within these well-established arteriopathy and connective tissue disorder genes may underlie non-syndromic SCAD susceptibility risk by increasing arterial fragility.

In addition to rare co-segregating variants, GWAS have implicated common risk-conferring variants and FBN1, LRP1, PHACTR1, ECM1, ADAMTSL4, LINC00310, C1orf54, MRPS21, and AFAP1 as candidate genes for SCAD [4,5,6], highlighting the heterogeneity of the disorder. Notwithstanding, we previously identified high-confidence second-degree protein–protein interactions between TLN1 and PHACTR1, FBN1 and LRP1 [5]. Moreover, among the 11 F-SCAD candidate-gene-encoded proteins identified in the current study, 10 had direct or indirect functional associations with TLN1 and/or common-risk gene-encoded proteins FBN1, LRP1, EDN1, and AFAP1 responsible for regulating cell adhesion, migration, and signaling pathways that orchestrate organization of the actin cytoskeleton [41,42,43,44,45]. These interactions suggest that both rare and common risk-conferring SCAD genes can have a shared role in regulation of the actin cytoskeleton, and variants within these genes may underlie SCAD pathogenesis.

Aberrant protein–protein interactions and shared pathways have been implicated in intracranial and thoracic aortic aneurysms and dissections [46,47]. In one family (SCAD-01), Val3698Glu substitution was identified in LRP2, a gene involved in coronary artery morphogenesis. The LRP2-encoded protein has a secondary interaction with two familial gene-encoded proteins (NOTCH1 and CTNNB1). LRP2, a major receptor of lipocalin-2, promotes angiogenesis through iron- and reactive-oxygen-species-related pathways in rodent endothelial cells [48].

Ten F-SCAD candidate-gene-encoded proteins had interactions with TLN1 and/or another familial gene-encoded protein and shared processes involved in cell adhesion. The formation and disassembly of adhesions through the binding of a cell to the extracellular matrix or other cells is essential for maintaining and repairing tissue architecture. Aberrant changes in cell adhesion dynamics have played a crucial role in the pathobiology of thoracic aortic dissections and aneurysms [49,50]. Interactions between adhesion complexes and actin, microtubules and intermediate filaments, elements that make up cytoskeleton networks, profoundly influence cell shape, motility, and remodeling [51].

### 4.1. Cell–Cell Adhesion

A Ser701Pro substitution in CDH4 was identified in SCAD-06. CDH4 is important for retinal vascular development [52]. CDH4 belongs to the type I classical cadherins responsible for mediating calcium-dependent adheren junctions [53]. Cell–cell junctions allow the cell to respond to changes within their microenvironment through connections with the actin cytoskeleton [54]. Moreover, within endothelial cells, these junctional complexes are responsible for regulating blood vessel maintenance and angiogenesis [55]. In SCAD-12, a Asn287Ser substitution was identified in CTNNB1, which is primarily responsible for regulating and coordinating cell–cell adhesion by anchoring cadherins to the actin cytoskeleton and involved in Wnt signaling [56]. Inactivation of Ctnnb1 in murine endothelial cells leads to a decrease in cell–cell adhesions and alterations in vascular morphogenesis [57]. Moreover, in rat carotid arteries, CDH4 expression and CTNNB1 signaling were associated with increased cyclin D1 expression and vascular smooth muscle proliferation, implicating both in vascular disease [56]. IQGAP1 is involved in regulating the cytoskeletal architecture through interactions with actin and microtubules [58]. Within this gene, we identified an Asn1066Asp substitution in SCAD-04. Acting as a scaffolding protein, IQGAP1 interacts with both catenins and cadherins and regulates cadherin-mediated cell–cell adhesions [59]. Further, IQGAP1 directly interacts with active vascular endothelial growth factor receptor-2, essential in endothelial migration and proliferation contributing to angiogenesis [60]. 

### 4.2. Cell–Extracellular Matrix Adhesion

A particularly notable finding in our study was the identification of collagen genes as top candidates for F-SCAD in five families (SCAD-09, -03, -07, -14, -15), each harboring a rare missense variant leading to substitution of a highly conserved amino acid: COL3A1 (Arg446Cys), COL4A1 (Pro530Ser), COL4A2 (Gly151Ser and Gly413Arg), and COL5A2 (Gln902Pro). While only three of the family members affected with SCAD had undergone formal evaluation by a medical geneticist (SCAD-03 proband; SCAD-07 proband and her sister), none had been suspected of Marfan syndrome, Ehlers–Danlos syndrome, or other multi-system CTD. Moreover, brain imaging performed in all but one affected family member (SCAD-09 proband) revealed subtle vascular disease in only one individual with a tiny cerebral aneurysm (SCAD-07 proband), whose maternal aunt also had a cerebral aneurysm by history. 

Collagens are a large family of extracellular matrix proteins responsible for a variety of functions, including tissue scaffolding, cell adhesion, and cell migration [61]. Collagens contain three polypeptide (α) chains that make up a triple helix, with a repeating Gly-X-Y triplet, in which every third residue is a glycine [61]. X and Y positions are frequently proline and 4-hydroxyproline residues that provide stability to the triple helix [61]. Each of our F-SCAD collagen variants were located within or near a triple helix region, and three were glycine or proline residue substitutions (Gly151Ser, Gly413Arg, and Pro530Ser). At the center of a triple helix where the α chains come together, a glycine residue is essential, as space precludes any other larger residue from fitting within the region [62]. Collagens have been associated with a wide spectrum of diseases. A large number of deleterious variants have been found in 22 collagen genes that range from early lethal phenotypes to common risk-conferring variants [62]. Included in heritable collagen disorders are Ehlers–Danlos syndrome (COL3A1, COL5A2) [63,64], brain small vessel disease-2 (COL4A2) [65], and arterial aneurysms (COL3A1, COL4A1) [66,67]. Of note, COL3A1 was one of seven genes with pathogenic variants identified in the first large-scale WGS study of sporadic SCAD [7]. Moreover, enrichment of rare variants in collagen genes has been reported subsequently in three independent WES and WGS studies of sporadic SCAD [36,37,38].

### 4.3. Adhesion Signaling

Cell adhesive interactions and networks that exist between both cell–cell and cell–matrix adhesions are controlled by signaling networks. Signaling networks are responsible for regulating downstream functions that include cell adhesion, migration, and mechanotransduction, thereby integrating signals from the outside and within the cell [68]. In EPHB4, we identified a Gly462Arg substitution in SCAD-05. EPHB4-mediated signaling, by activation from ephrinB2 ligand, is involved in regulating cell adhesion and migration [69]. Inactivation of Ephb4 in murine endothelial cells leads to rupturing of cardiac capillaries and cardiomyocyte hypertrophy [70]. An Arg1279Cys substitution in NOTCH1 was identified in SCAD-13, one of two instances in our study of a likely pathogenic variant based on ACMG criteria. Moreover, NOTCH1 fulfilled five secondary parameters in our filtering workflow. NOTCH1 receptor signaling is involved in a wide variety of processes, including regulating adheren junction assembly and vascular barrier function [71]. Deleterious variants within NOTCH1 have been associated with bicuspid aortic valve disease, thoracic aortic aneurysms, and pulmonary hypertension [72,73]. In a recent case report, a Arg1438Cys substitution was identified by WES in a patient with recurrent and pregnancy-associated SCAD [74].

An Arg261fs* deletion in KCNK3 was identified in SCAD-02, the second instance of a likely pathogenic variant. KCNK3 is responsible for regulating K+ conductance and resting membrane potential in pulmonary artery smooth muscle cells [75]. Deleterious variants within KCNK3 have been linked to pulmonary arterial hypertension [76].

### 4.4. Limitations

The co-segregation filter proved to be a powerful tool for generating relatively short lists of potential SCAD-susceptibility genes in each family. However, the families in this study were not powered for genome-wide linkage analysis; co-segregation with SCAD was inferred in two families (SCAD-09, SCAD-15), for which clinical and genetic data were lacking in the relative with SCAD, and our candidate-gene-ranking workflow imposed strict gene-constraint requirements and relied on existing bioinformatics databases, predictive metrics, and known protein–protein and gene–disease associations. Consequently, additional co-segregating variants could have a role in SCAD pathogenesis. Predicting the potential biological impact of non-coding region variants poses a particular challenge. Functional experiments and identification of additional rare coding or non-coding variants in top candidate genes within other SCAD cohorts would lend further support to our findings.

## 5. Conclusions

F-SCAD is a genetically heterogeneous disorder, yet collagen genes were top candidates in five families in our cohort. Moreover, several genes had high-confidence primary or secondary protein–protein interactions with both TLN1 and FBN1, and our study further implicates perturbation of structural proteins that function in actin cytoskeleton and cell–cell adhesion in disease pathogenesis. The well-established predilection of SCAD for women was also observed in most of our F-SCAD kindreds. However, our top-ranked candidate genes did not exhibit sex-specific differences in coronary artery mRNA expression, suggesting that risk allele effects may be accentuated by a generalized increase in vascular fragility conferred by female hormone milieu [77]. Indeed, incomplete penetrance and variable expression of rare, predicted-deleterious, co-segregating variants implicate genetic or environmental modifiers of this episodic disorder.

## Figures and Tables

**Table 1:** Familial SCAD demographics and clinical characteristics.

Family	ID	Sex	Race	Age at Event/ Diagnosis, Yrs	Clinical Features	Affected Coronary Artery
SCAD-01	II.1	F	W	42	SCAD, MH	MV: LM, LCx, OM2, D1
	II.2	F		47	SCAD, FMD, P, ES	OM
SCAD-02	II.1	F	W	35	SCAD, CT, ES	LAD
	II.2	F		45	SCAD	LAD
SCAD-03	II.1	F	W	43	SCAD, FMD, CT	LAD
	II.2	F		45	SCAD, MH	LAD
SCAD-04	II.1	F	W	43	SCAD, ES	MV: LCx, OM
	II.2	F		59	SCAD	LAD
SCAD-05	II.1	F	W	34	SCAD, FMD, P, PE	OM2
	II.2	F		36	SCAD, FMD	LAD
SCAD-06	II.1	F	W	50	SCAD, FMD, ICAA	MV: LAD, LCx
	II.2	M		47	SCAD	DA
	II.3	M		40	SMAD	—
SCAD-07	III.1	F	W	41	SCAD, FMD, ES, CA	LAD
	III.2	F		37	SCAD, FMD, CT	LAD
	II.3	F		65	CA, ICAA	—
SCAD-08	III.1	F	W	50, 59	SCAD, R, ES	1. LAD, 2. OM1
	III.2	F		50	SCAD, CT	OM1
	I.1	M		71	CA	—
SCAD-09	III.2	F	W	42	SCAD, ES	MV: RCA, PDA
	III.3	F		Unk	SCAD, FMD	
	III.1	M		25	CVA	—
SCAD-10	II.3	F	W	42, 45, 56	SCAD, R, ES	1. LCx, 2. MV: LAD, LM, 3. RCA
	III.1	F		39	SCAD, MH	LAD
	II.2	F		Unk	CT	—
SCAD-11	III.1	F	W	36	SCAD, FMD	OM1
	III.2	F		44	SCAD, CT, MH, PE	RCA
SCAD-12	II.3	F	W	47	SCAD, FMD	LCx
	III.1	F		38	SCAD, P	OM
	II.1	F		Unk	CA	—
SCAD-13	II.1	F	W	44	SCAD	LAD
	I.2	F		54	SCAD	LAD
	II.2	F		45	CD	—
SCAD-14	I.2	F	W	59, 61	SCAD, FMD, R	1. MV: LCx, OM2, 2. LAD
	II.1	F		32	SCAD, PE	LAD
SCAD-15	II.1	F	W	33	SCAD, FMD, MH	LAD
	II.2	F		Unk	SCAD	
	I.1	F		65	AAA, IAA, PAA	—

AAA, abdominal aortic aneurysm; CA, cerebral aneurysm; CD, carotid artery dissection; CT, coronary tortuosity; CVA, cerebrovascular accident; DA, diagonal artery; ES, emotional stress; F, female; FMD, fibromuscular dysplasia; ICAA, internal carotid artery aneurysm; IAA, iliac artery aneurysm; LAD, left anterior descending coronary artery; LCx, left circumflex coronary artery; LM, left main coronary artery; M, male; MH, migraine headache; MV, multivessel; OM, obtuse marginal artery; P, pregnancy-associated; PAA, popliteal artery aneurysm; PE, physical exertion; R, recurrent; RCA, right coronary artery; SMAD, superior mesenteric artery dissection; and W, white.

**Table 2:** Top candidate genes in familial SCAD. Minor allele frequencies are based on all populations. AA, aortic aneurysm; Aor, aortopathy; AAA, abdominal aortic aneurysm; AD, aortic dissection; CAC, coronary artery calcification; CAD, coronary artery disease; CADD, combined annotation-dependent depletion; ceAD, cervical artery dissection; CoA, coarctation of the aorta; CTD, connective tissue disorder; CTHD, conotruncal heart defects; CSVD, cerebral small vessel disease; CVD, cardiovascular disease; gnomAD, Genome Aggregation Database; GWAS, genome-wide association study; HGMD, Human Genome Mutation Database; IA, intracranial aneurysm; ICH, intracerebral hemorrhage; LVOTO, left ventricular outflow tract obstruction; MGI, Mouse Genome Informatics; PAH, pulmonary arterial hypertension; TAD, thoracic aortic disorder; TGA, transposition of the great arteries; TOF, tetralogy of fallot; VAD, intracranial vertebral artery dissection.

Gene Symbol (Name)	rsID	Variant	Translation Impact	gnomAD MAF (%)	CADD Score (Percentile)	Deleterious Classification (HGMD)	Gene Constraint (Z-Score/pLI ^†^)	Arterial Tissue Expression—Rank			PPI Talin 1 = 1, GWAS = 2	CTD/ Aor Gene	Abnormal Vascular Phenotype (MGI)	Vascular Phenotype (HGMD)	GWAS Trait (GWAS Cat)
								Coronary	Aorta	Tibial					
*CDH4* (cadherin 4)	rs1305825960	c.2101T > C p.S701P	Missense	0.0004	25.1 (75)	—	2.02	47	46	43	2	—	—	—	CAC, ICH
*COL3A1* (collagen type III alpha 1 chain)	rs1238066761	c.1336C > T p.R446C	Missense	0.0004	32 (95)	—	4.09	6	5	7	1, 2	Yes	AD, aorta smooth muscle morphology	TAD, AA, CSVD, AAA, SCAD	—
*COL4A1* (collagen type IV alpha 1 chain)	rs145172612	c.1588C > T p.P530S	Missense	0.03	29.4 (90)	CM1818194	3.02	1	3	2	1, 2	—	ICH, retinal vascular morphology	ceAD, CSVD, ICH, CAD, TGA	Arterial stiffness, CVD, CAD
*COL4A2* (collagen type IV alpha 2 chain)	rs746743018	c.451G > A p.G151S	Missense	0.002	24.8 (75)	—	2.19	2	1	3	1, 2	—	Aorta stenosis, cranial blood vascular morphology	CSVD, ICH	Arterial stiffness, carotid artery thickness, CAC, CAD
	rs1464563247	c.1237G > A p.G413R	Missense	0.001	26.6 (85)	—									
*COL5A2* (collagen type V alpha 2 chain)	—	c.2705A > C p.Q902P	Missense	NR	27.5 (90)	—	2.44	6	4	8	1, 2	Yes	Vascular congestion, cardiovascular system physiology	ceAD, CTHD, AD, IA, SCAD	—
*CTNNB1* (catenin beta 1)	rs35288908	c.860A > G p.N287S	Missense	0.09	21.2 (30)	CM043757	3.85	13	15	3	1, 2	—	Pulmonary artery, vascular endothelial cell and vitelline morphology	—	—
*EPHB4* (ephrin type-B receptor 4)	rs146674844	c.1384G > A p.G462R	Missense	0.03	24.8 (75)	—	2.30	30	37	38	—	—	Angiogenesis, vascular branching, pulmonary artery and vitelline morphology	PAH, LVOTO	—
*IQGAP1* (IQ-motif-containing GTPase-activating protein1)	—	c.3196A > G p.N1066D	Missense	NR	27.7 (90)	—	2.44	5	4	3	1, 2	—	—	TOF and other cardiac abnormalities	—
*KCNK3* (potassium channel subfamily K member 3)	—	c.781_794delCGCGCGCTGCTCAC p.R261fsTer	Frameshift *	NR	—	—	0.90 ^†^	5	13	7	—	—	—	PAH	CVD, ICH
*LRP2* (LDL-receptor-related protein 2)	—	c.11093T > A p.V3698E	Missense	NR	24.6 (75)	—	2.07	35	31	34	2	—	Aortic arch morphology	PAH	—
*NOTCH1* (neurogenic locus notch homolog protein 1)	rs182330532	c.3835C > T p.R1279C	Missense *	0.06	34 (99)	—	3.45	26	28	22	1, 2	Yes	Angiogenesis, vasculogenesis, vitelline morphology	TAD, PAH, VAD, CoA, TOF, ceAD	CAC

* Predicted likely pathogenic by American College of Medical Genetics and Genomics criteria. ^†^ pLI, probability of loss of function intolerance score.

## Data Availability

The data from this study are available from the corresponding author upon reasonable request.

## Supplementary Materials

The following supporting information can be downloaded at https://www.mdpi.com/article/10.3390/jcdd10090393/s1. The following supporting information can be found in the Supplementary File, Table S1: Top candidate genes harboring non-coding variants in familial SCAD. Figure S1: Non-coding variant filtering and gene prioritization workflow identifies additional candidate genes for familial SCAD. Figure S2: Non-coding variants in familial SCAD alter transcription factor and microRNA binding sites [78,79]. Figure S3: Functional association between individual F-SCAD non-coding candidates and previously reported SCAD genes. Figure S4: Network interactions of functional associations among F-SCAD non-coding and coding candidates.

## Abbreviations

ACMG, American College of Medical Genetics; CTD, connective tissue disorder; F-SCAD, familial SCAD; FMD, fibromuscular dysplasia; GWAS, genome-wide association study; HGMD, Human Gene Mutation Database; SCAD, spontaneous coronary artery dissection; WES, whole-exome sequencing; WGS, whole-genome sequencing.
