# Supplementary material for: Identification of Rare Genetic Variants in Familial Spontaneous Coronary Artery Dissection and Evidence for Shared Biological Pathways
Source: J Cardiovasc Dev Dis. 2023 Sep 12;10(9):393. doi: 10.3390/jcdd10090393 (PMC10532385; doi:10.3390/jcdd10090393)
Supplement: Supplementary file 1 [file jcdd-10-00393-s001.zip › jcdd-2579832-supplementary.pdf]

SUPPLEMENTAL MATERIAL

**Gene Symbol** (Name)	**rsID**	**Variant**	**Regulatory Site**	**gnomAD MAF** (%)	**CADD Score** (percentile)	**FATHMM-XF** (Non-coding pathogenic)	**Arterial Tissue Expression- Rank**			**PPI** Talin 1=1, GWAS=2	**CTD/Aor Gene**	**Abnormal Vascular Phenotype** (MGI)	**Vascular Phenotype** (HGMD)	**GWAS Trait** (GWAS Cat.)
							**Coronary**	**Aorta**	**Tibial**					
ATF4 (Activating transcription factor 4)	rs571672362	c.-277 C>T	TFBS	0.099	13.3 (90)	0.709	5	4	2	2	–	aorta morphology	–	–
PDGFRA (Platelet derived growth factor receptor alpha)	rs184179322	c.*2496 A>G	miRNA binding	0.022	20.9 (95)	0.834	9	6	22	1, 2	–	placenta vasculature, vasculogenesis, vitelline morphology	aortic arch anomaly	–

Table S1. Top candidate genes harboring non-coding variants in familial SCAD. Minor allele frequencies are based on all populations. Aor, aortopathy; CAD, coronary artery disease; CADD, combined annotation-dependent depletion; CTD, connective tissue disorder; CVD, cardiovascular disease; FATHMM, functional analysis through hidden Markov models; gnomAD, Genome Aggregation Database; GWAS, genome-wide association study; HGMD, Human Genome Mutation Database; miRNa, microRNA; MGI, Mouse Genome Informatics; PAH, pulmonary arterial hypertension; PPI, protein-protein interaction; TFBS, transcription factor binding sites. Asterisk denotes a 3’ untranslated region.

			SCAD-08 n=2	SCAD-10 n=2	SCAD-11 n=3
Primary	Variant	**High Confidence**	6,116,253	5,175,253	5,622,361
		**Rare** - MAF <0.1%	687,345	260,161	404,778
		**Segregates with Arteriopathy**	106,432	30,532	17,126
		**Regulatory Impact** (TFBS or miRNA coding/ binding site)	6	8	5
		**Regulatory** (CADD ≥7)	3	4	3
		**RegulomeDB** (Ranking ≤3)	4	5	5
		**FATHMM-XF** (Non-coding pathogenic score)	**1**	**1**	**0**
Secondary	Gene	**Arterial Tissue Expression** OR	0	*ATF4*	
		**Overlapping Gene** OR	0	0	
		**Protein-Protein Interaction** OR	*PDGFRA*	0	
	Gene-Disease Association	**CTD/Aortopathy Gene** OR	0	0	
		**Mouse Vascular Phenotype** OR	*PDGFRA*	*ATF4*	
		**Human Vascular Phenotype** OR	*PDGFRA*	0	
		**GWAS Vascular Trait**	0	0	
		**Prioritized Candidate Gene**	***PDGFRA***	***ATF4***	—

Figure S1. Non-coding variant filtering and gene prioritization workflow identifies additional candidate genes for familial SCAD. A non-coding variant filtering scheme applied to all single-nucleotide variants and insertions/deletions in families SCAD-08, SCAD-10 and SCAD-11 identified two genes in two families. Abbreviations: CADD, Combined Annotation Dependent Depletion; CTD, connective tissue disorder; GWAS, genome-wide association study; MAF, minor allele frequency; pLI, probability of loss-of-function; SCAD, spontaneous coronary artery dissection.

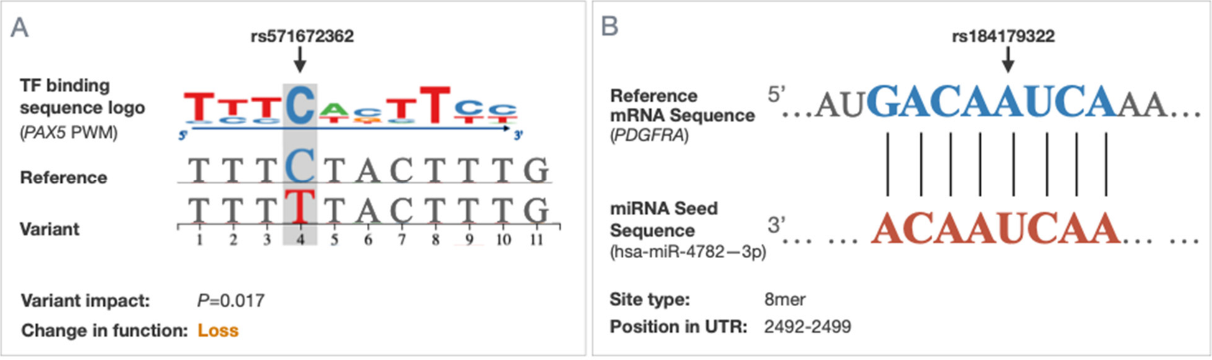

Figure S2. Non-coding variants in familial SCAD alter transcription factor and microRNA binding sites**. A,** A composite logo plot (represented with position weight matrices – letter heights indicate sequence conservation at that position) of a binding site within the 5 'untranslated region of the non-coding candidate *ATF4* for the transcription factor *PAX5*. The rs571672362-T variant is predicted to cause a loss of function and prevent binding of *PAX5* according to atSNP [78]. **B,** A predicted microRNA binding site and seed sequence of miR-4782-3p overlapping rs184179322 within the non-coding familial candidate *PDGFRA* [79].

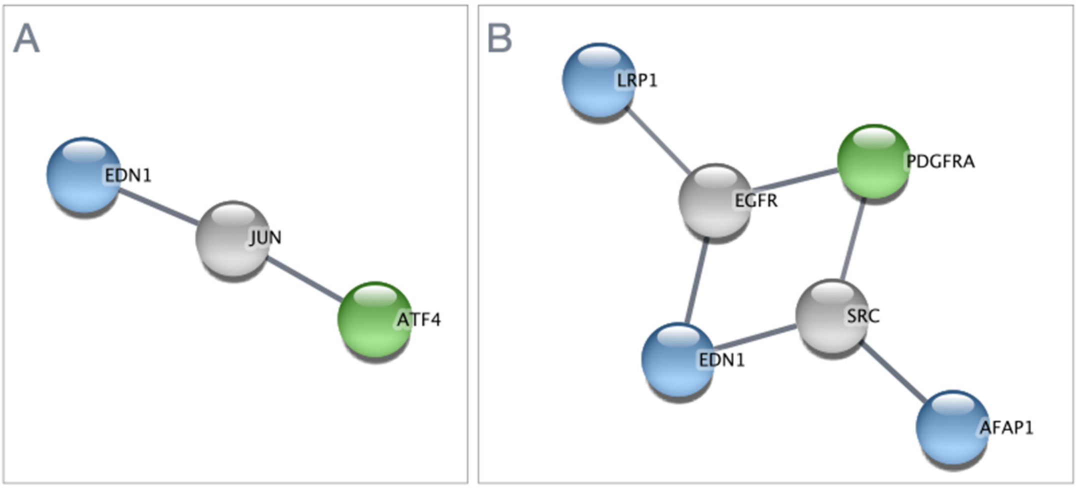

Figure S3. Functional associations between individual F-SCAD non-coding candidates and previously reported SCAD genes. GWAS-derived candidate gene proteins EDN1, LRP1, and AFAP1 (blue) interact with two of the prioritized non-coding candidates (Panel A, ATF4; Panel B, PDGFRA) for familial SCAD (green) through a STRING 2nd shell interactor protein (grey). STRING analysis was set at a high confidence score (0.7).

A

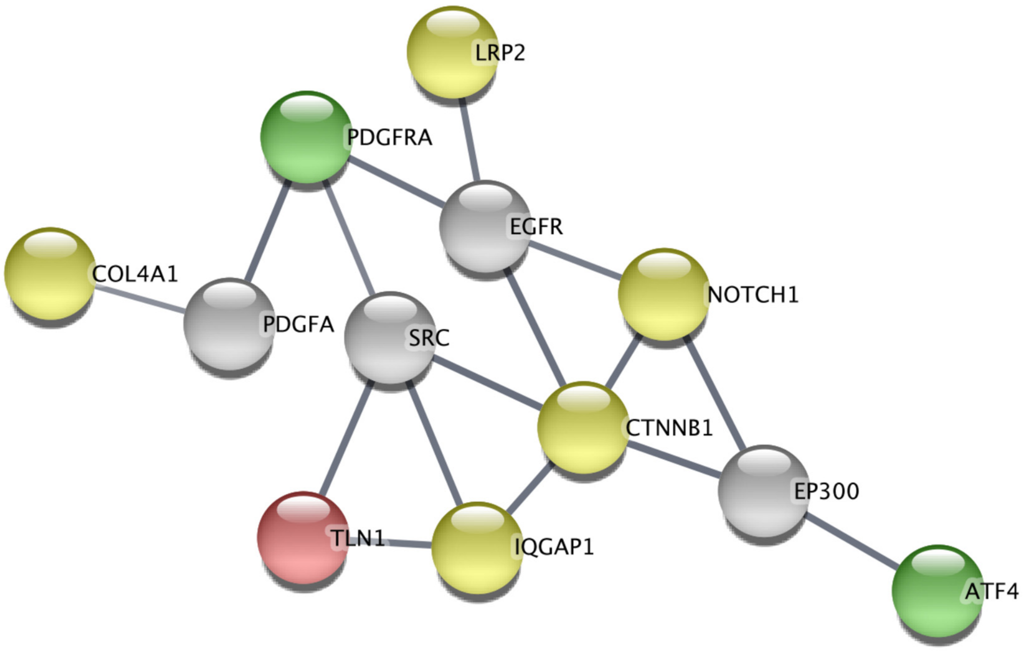

B

Protein-Protein Interaction	Confidence Score	Textmining	Database	Coexpression	Experiments
ATF4_EP300_CTNNB1	0.969/0.999	0.432/0.989	0.800/0.900	—/0.054	0.753/0.897
ATF4_EP300_NOTCH1	0.969/0.997	0.432/0.906	0.800/0.900	—/0.062	0.753/0.678
PDGFRA_PDGFA_COL4A1	0.999/0.772	0.976/0.282	0.900/0.600	—	0.875/0.270
PDGFRA_SRC_TLN1	0.849/0.974	0.517/0.609	0.800/0.900	0.062/0.083	0.122/0.270
PDGFRA_SRC_IQGAP1	0.849/0.966	0.517/0.561	0.800/0.900	0.062/0.062	0.122/0.270
PDGFRA_SRC_CTNNB1	0.849/0.999	0.517/0.978	0.800/0.900	0.062/0.060	0.122/0.678
PDGFRA_EGFR_LRP2	0.958/0.920	0.747/0.211	0.800/0.900	0.098/0.063	0.642/0.057
PDGFRA_EGFR_NOTCH1	0.958/0.834	0.747/0.756	0.800/—	0.098/—	0.642/0.229
PDGFRA_EGFR_CTNNB1	0.958/0.999	0.747/0.984	0.800/0.900	0.098/0.062	0.642/0.715

Figure S4. Network interactome of functional associations among F-SCAD non-coding and coding candidates. **A,** Protein-protein interactions in two of the familial non-coding prioritized candidates (green) and six of the familial coding candidate (yellow) and talin 1 (red) through a STRING 2nd shell interactor (grey). **B,** Shared protein-protein interactions had a high confidence score of <0.8.

Supplemental References

78. Zuo, C.; Shin, S.; Keles, S. atSNP: transcription factor binding affinity testing for regulatory SNP detection. *Bioinformatics*. **2015**, 31, 3353-3355, doi:10.1093/bioinformatics/btv328.

79. McGeary, S.; Lin, K.; Shi, C.; Pham, T.; Bisaria, N.; Kelley, G.; Bartel, D. The biochemical basis of microRNA targeting efficacy. *Science*. **2019**, 366, eaav1741, doi:10.1126/science.aav1741.
